# Work-life balance and self-reported health among working adults in Europe: a gender and welfare state regime comparative analysis

**DOI:** 10.1186/s12889-020-09139-w

**Published:** 2020-07-16

**Authors:** Aziz Mensah, Nicholas Kofi Adjei

**Affiliations:** 1 Bielefeld Graduate School in History and Sociology (BGHS), Bielefeld University, Universitätsstrasse 25, 33615 Bielefeld, Germany; 2 Leibniz Institute for Prevention Research and Epidemiology – BIPS, Bremen, Germany; 3 Health Sciences Bremen, University of Bremen, Bremen, Germany

**Keywords:** Work-life balance, Self-reported health, Gender, Welfare states, Working adults

## Abstract

**Background:**

The pressing demands of work over the years have had a significant constraint on the family and social life of working adults. Moreover, failure to achieve a ‘balance’ between these domains of life may have an adverse effect on their health. This study investigated the relationship between work-life conflict and self-reported health among working adults in contemporary welfare countries in Europe.

**Methods:**

Data from the 6th European Working Conditions Survey 2015 on 32,275 working adults from 30 countries in Europe were analysed. Multivariate logistic regression models were used to examine the associations between work-life balance and self-reported health among men and women. We further used a 2 stage multi-level logistic regression to assess variations in self-reported health among welfare state regimes by gender.

**Results:**

The results showed a strong association between work-life conflict and poor self-reported health among working adults in Europe (aOR = 2.07; 95% CI: 1.93–2.23). However, the magnitude of the effect differed slightly by gender (men: aOR = 1.97; 95% CI: 1.78–2.18 vs women: aOR = 2.23; 95% CI: 2.01–2.47). Furthermore, we found variations in the relationship between work-life conflict and poor self-reported health between welfare states regimes. The association was found to be weaker in the Nordic and Southern welfare states than the Liberal, Conservative, and Central Eastern European welfare states. Although the associations were more consistent among men than women in the Conservative welfare states regime, we found higher associations for women than men in the Southern, Nordic, Liberal, and Central Eastern European welfare states.

**Conclusions:**

This study provides evidence of some variations in the association between work-life conflict and poor self-reported health among men and women across welfare states regimes in Europe. The results demonstrate the need for governments, organizations and policymakers to provide conducive working conditions and social policies for working adults to deal with competing demands from work and family activities.

## Introduction

The changing patterns of work over the years have had a significant constraint on both the family and the social life of working adults [1]. With the limited 24 h’ time resource available in a day, working adults may be confronted with many challenges, including deadlines to meet targets, financial obligations, and pressing family responsibilities. These situations may create role conflict, which can affect the level of involvement in their work, family and social life [2–4]. Some scholars suggest that higher demands from household activities make it difficult to balance work and family life [2, 3]. A recent study on work-life conflict among employees in Europe revealed a work-life ‘imbalance’ among employees in Europe [5]. This phenomenon has partly been attributed to an increase in the involvement of women in the labour force and the rising involvement of men in performing housework, including child care and family chores [6, 7]. The Evidence further suggests that time allocated by men to housework activities has increased over time [8, 9], while female participation in the labour market has also increased over the years [10]. Work-life conflict may arise when there is a role conflict in the satisfaction of work and family life [11], and failure to achieve a ‘balance’ between these domains may have an adverse effect on working adults’ health [5, 12, 13].

Work-life conflict among employees is known to be related with many health problems, including poor physical health [14–16], poor-self reported health [15, 17], psychological distress [14, 18], poor mental health [19–21] and life dissatisfaction [22]. However, work-life conflict and health outcomes may differ by gender due to the unequal distribution of work-related roles [23–27]. For example, some previous studies found a positive relationship between work-life conflict and poor self-reported health among working women than men [25–27], while other studies suggest similar outcomes between men and women [5, 28]. In a longitudinal study among working adults in Sweden, Leineweber et al. [27] found an association between work-life conflict and suboptimal self-reported health among working women than men. Similarly, Eek and Axmon [26] found that women in relationship with unequal distribution of work and family activity reported a higher level of fatigue, stress, and physical symptoms than those in relationship with equal distribution of responsibility. In contrast, Kinnunen and his colleagues [28] found no evidence of gender difference in the association between poor work-life balance and health outcomes such as life satisfaction and well-being.

### Gender, work life balance, and welfare policies

Gender plays a key role in understanding how work and other domains of life are distributed and performed [29]. The term is not static but rather a phenomenon where identity is continuously renegotiated [30, 31]. Evidence suggests that traditional and societal expectation of behaviour differs between men and women [32, 33], where women are responsible for caregiving (family activities) and other household activities, while men assume the primary role for paid work activities [10, 34]. For example, using time use data, Aliaga [35], Hagqvist [36] and Adjei et al. [37] indicated that women spent more time on family activities than men, while men spent more time on paid work activities than women. Similarly, research conducted by Boye [38] revealed that about 40% of working-age women are not engaged in paid work activities as compared to men (1.5%). The study further showed that women spend about 13 h more on unpaid work per week as compared to men. Hochschild [39] argued that although women’s contribution and participation in paid work activities have dramatically increased over the years, it has not been accompanied by a proportionate measure of increase in time allocation to unpaid work by men. Women continue to spend more time on household activities as compared to men [40]. However, recent studies suggest that women have reduced their time and involvement in unpaid work while men have increased the amount of time devoted to unpaid work activities [9], especially child care [8, 9]. Kan et al. [41] argued that the change in the reduction of time spent on household activities by women could be attributed to the increase of women in the labour market rather than a change of ideology among men in the performance of household activities.

The rational view proposed that work-family conflict will increase when there is an increase in the amount of time spent on both work and family activities [32]. This phenomenon has been attributed to role strains [42]. In their study, Frone et al. [15] noted that long working hours, psychological involvement in work, inflexible working time arrangement, lack of clarity of work function, and role overload are indicators that influence work-life conflict among employees. There have been many studies on gender and work-life conflict [33, 43, 44]; however, the findings from these studies are inconclusive and contradicting [45]. While some studies found higher work-life conflict among women than men [33, 43, 44], few studies failed to demonstrate any significant difference among men and women [46–48]. In a cross-sectional study in the UK, Emslie et al. [46] found that both white-collar men and women employees in the Bank have the same level of work-family interference. Similarly, Schiemann et al. [47] found no gender difference in work-life conflict among higher status workers in Canada. These authors attributed their findings to the egalitarian gender role balance that suggest that the level of expectation in terms of sharing financial and family responsibilities is similar for men and women [47]. Nonetheless, using longitudinal data from the German Socio-Economic Panel Study (SOEP), Busch-Heizmann and Holst [44] found a higher prevalence of interference between work and family life among working women than men in Netherland. Gutek et al. [33] attributed some of the reasons to the fact that women still retain their primary role of performing care and other household activities even when they are confronted with higher job demands. Men, on the other hand, are more satisfied when they devote more time and effort to paid work- than household activities [49]. Ngo and Lui [45] also suggested that work- life conflict is higher among women due to limited control over conflicting domains of life. It has also been established that women who are affected by work-life conflict may experience higher forms of stress and other adverse health outcomes than men [33, 43].

Gender inequality in work-life conflict has also been linked with socio-economic policies that exist within countries [50]. According to Gornick [51], extensive parental leave, support for childcare and elderly care, strong labour regulation, and universal health service that exist within countries are factors that may influence interference between work and family life. The development of welfare policies may also be rooted in historical, social, and economic development that exist in a country [52]. Hence, there may be variations among countries in terms of generosity, focus, and goals of social and welfare policies [53]. Contemporary welfare policies may be classified into five distinct regimes, namely, Nordic (social democratic), Liberals (Anglo-Saxon), Conservative (Corporatist), Southern Europe, and Central Eastern Europe (CEE) [54]. Esping-Andersen [53] described countries in the Nordic welfare states regime as having policies that are ‘encompassing,’ where the level of social support is generous and universal. This type of welfare system encourages dual-earner family roles, extensive support to single parents, and a regulated labour market, which allows more women to participate in the labour market [55]. In addition, there is provision for publicly funded child and elderly care services [55, 56], and extensive paid parental leave days for working women and men [57]. Liberal welfare states, on the other hand, are characterized by a strong male-breadwinner model with childcare primarily provided by a private venture with low state support [58]. These countries are also characterized by weak employment regulations and less generous state provision of social services and benefits [53, 59]. Conservative welfare states are characterized by traditional male breadwinner family models and have strong labour market laws to regulate employment [53]. In this regime type, families bear the responsibility for primary social welfare benefits [59], and most working mothers engage in part-time or secondary jobs without good economic remuneration [60]. In the Southern European welfare states regime, social benefits are much lower [59, 61] as compared to the Conservative welfare states regime. Moreover, care services are largely provided by family, friends, and volunteers. Familialism is stronger in this regime type [39], and there are gender roles, where men are known to be ‘breadwinners’ and women as ‘caregivers’ [39]. The CEE welfare states are also characterized by the dual-earner family model but weak trade unions and labour regulations [62], and a traditional division of housework [63].

Welfare policies may influence work-life balance and might subsequently have an effect on health outcomes [5, 14, 15, 64]. Countries with more generous social policies such as quality child care service, extensive parental leave, and generous social benefits may influence the magnitude of the association between work-life conflict and health-related outcomes [5, 64, 65]. For instance, Artazcoz and his colleagues [64] could not find any evidence that work-life conflict and poor self-reported health among working adults in northern Europe, where many generous welfare policies exist, but the study found an association between work-life conflict and poor health in Conservative and Southern European welfare states with less generous welfare benefits. In contrast, Hagqvist and his colleagues [12] noted that Nordic countries may show higher association between work-family conflict and low well-being as compared to countries with a more traditional family model in Europe.

A plethora of studies on work-life balance and health status have been based on a single country [25, 41, 66]. Still, only a few studies have focused on cross-country variation in welfare state typologies as well as gender differences [9, 67]. To the best of our knowledge, no previous study has used a more comprehensive cross-country sample as the underlying conceptual structure for making a comparison. Hence, this study seeks to contribute to a deeper understanding of the gender difference in the relationship between work-life conflict and poor self-reported health among working adults in Europe. In addition, we analyse whether these effects vary across different welfare state regimes in Europe. By drawing on the theoretical relationship that exists between work-life conflict and self-reported health, the following research questions will be addressed:

1. Is there a relationship between work-life conflict and poor self-reported health among working adults in contemporary welfare states in Europe?
2. Does the relationship between work-life conflict and poor self-reported health differ by gender?
3. To what extent do these relationships vary by welfare state regimes among men and women in Europe?

## Methods

### Data

This study was based on the 6th European Working Conditions Survey (EWCS 2015), conducted by the European Foundation for the Improvement of Living and Working Conditions. The EWCS survey data covered 35 countries in Europe. This includes EU28 countries, two countries from the European Free Trade Association (Norway and Switzerland), and five potential EU candidates’ countries (Albania, the Former Yugoslav Republic of Macedonia, Turkey, Serbia, and Montenegro). The target population of the survey was working adults who were between the ages of 15 years and above. The EWCS adopted a multistage, stratified, random sample in selecting the target population in each country. The target sample size for most countries was 1000, however, because some countries had larger workforce than others, the sample size varied [68]. For instance, the target sample size was increased to 1200 for Poland, 1300 for Spain, 1400 for Italy, 1500 for France, 1600 for UK, and 2000 for both Germany and Turkey. Furthermore, the European Foundation for the Improvement of Living and Working Conditions (Eurofound) also offered opportunity for countries to top-up their sample size. This opportunity was taken up by Belgium, Slovenia, and Spain which allowed them to increase their sample size to 2500, 1600, and 3300 respectively. Each country was stratified by region and the degree of urbanization. Primary sampling units (PSU) were randomly selected with probability proportional to size in each of the stratum. A random sample of household or individuals were further selected from each PSU [68]. A total of about 44,000 respondents were selected for a face-to-face interview in their respective households.

We restricted our analysis to working adults aged 16–64 years who were non-retired, not full-time homemaker, not a full-time student, and nondisabled. Respondents who refused to answer specific questions or do not know answers to specific questions were also excluded from the analysis. We also limited our study to 30 countries in Europe (i.e., the EU 28 countries, Switzerland and Norway). Missing responses were excluded because they accounted for less than 2% of the sample size. In the final analysis, we included a total of 32,275 participants.

### Measures

Self-reported health, our outcome variable of interest, was measured using the question, “How is your health in general?” Responses were rated from 1 (very good), 2 (good), 3 (fair), 4 (bad), 5 (very bad). Self-reported health has been shown to be a good proxy for measuring health status and a reliable technique as a predictor of mortality [69]. To avoid much-skewed distribution of responses [70], we dichotomized the responses as done in previous studies [5, 64, 71, 72]. Respondents who answered very good and good were categorized as having “good self-reported health”, while those who answered fair, bad, and very bad were categorized as having “poor self-reported health”. Our approach for dichotomizing the responses for the self-reported health was supported by existing research which mentioned that when five multiple options are available for a respondent to choose, the response that falls in the middle is closer to the negative responses as compared to the positive responses [73].

Work-life balance, our primary exposure of interest, was measured with the following question: “In general, how do your working hours fit in with your family or social commitments outside work?” Responses were: very well, well, not very well, and not at all well. To aid interpretability of our study, we further dichotomized the answers as good work-life balance (“very well” or, “well”) and poor work-life balance or work-life conflict (“not very well”, or “not at all well”).

The working characteristics of respondents were measured based on the Standard Industrial classification (NACE), sector, years of service, working arrangement, form of employment, type of employment, and weekly hours. NACE was classified into four categories (agriculture, industry, service, and other). Sector of employment was classified into five categories (private, public, joint private-public, NGO, and other). Shift work was measured with the question, “do you work shifts?” The responses were grouped as “Yes” or “No”. Working arrangement was categorised as (set by company, can choose between fixed schedule, flexible working time, working time is determined by self). We dichotomized the type of employment (employee and self-employed). Working hour was divided into five categories (30 h and below, 31–40 h, 41–50 h, 51–60 h, 60 h +). Regarding welfare regime types, we grouped countries according to common welfare state regime features. This study adopted Ferrera [74] and Bambra and Eikemo [75] classification of welfare typologies: Nordic (Sweden, Denmark, Finland, and Norway), Conservative (Austria, Belgium, France, Germany, Netherland, Luxembourg, and Switzerland), Liberals (United Kingdom and Ireland), Southern Europe (Greece, Spain, Italy, Portugal, Cyprus, and Malta), and Central and Eastern Europe (CEE) (Estonia, Lithuania, Hungary, Czech Republic, Poland, Latvia, Romania, Slovakia, Slovenia, Bulgaria, and Croatia).

Demographic characteristics including gender (male and female), household size, and age, marital status (single or widowed, married or cohabiting), and living with child (yes, no) were further explored. Socio-economic position was measured by education and occupation. Education was categorized in accordance with the International Standard Classification of Education-2011 (early childhood, primary, lower secondary, upper secondary, post-secondary, short cycle tertiary, bachelor, master, doctorate). The measurement of occupation was in line with the International Standard Classification of Occupation-08 (managers, armed forces, professionals, technicians and associate professionals, clerical support workers, agricultural workers, plant and machine operators, and elementary occupations).

### Analytical strategy

Descriptive statistics were used to describe the study population. Furthermore, a bivariate test was performed on the measured variables by gender. We adopted a chi-square test for categorical variables [76], and a point biserial correlation test for continuous variables [77]. Variables that were significantly associated with the outcome variable were selected to estimate the odds ratios. In order to determine the association between self-reported health and work-life balance, a multivariate logistic regression was applied, adjusting for socio-economic position, working conditions, and demographic characteristics. Odds ratios and 95% confidence interval for all models estimated were presented for analysis. In addition, we estimated the Variance Inflation Factor (VIF) to test for multicollinearity of the independent variable and the covariates. The VIF is a more superior technique in determining collinearity [78]. According to O’Brien [78], a threshold value of VIF < 10 is an indication of low multicollinearity or non-existence of multicollinearity. Due to the clustering nature of the sample, we extended our multivariate logistic regression to a multi-level logistic regression to examine variations between welfare regimes and by gender. A two-stage multi-level logistic regression was applied with individual working adults nested within welfare state regimes. This was done across welfare state regimes, where the strength of the associations was compared. Furthermore, we estimated the median odds ratio (MOR) and the variance partition coefficient (VPC). The VPC is the percentage of variation that may occur in higher levels (welfare state regimes) [79]. Similarly, the MOR quantifies the level of variations that may exist between countries in Europe [5]. If the MOR is equal to 1, then there is no variation between countries across welfare state regimes, however, if the MOR is larger than 1, then there is a variation between countries in Europe [80]. All analyses were performed using Stata V14 [81] and done separately for men and women.

## Results

### General distribution and sample characteristics

Table 1 provides information on the general descriptive statistics of working men and women of the 6th EWCS 2015. The mean age across welfare states regimes was quite similar among men and women. We observed good work-life balance among working men to be higher in the Nordic welfare states regime (85.6%), followed by the Conservative welfare states regime (82.0%). Women in the Nordic welfare states regime also had the highest (86.9%) frequency of good work-life balance, followed by the Conservative welfare states regime (85.1%). Furthermore, the highest proportion of poor work-life balance was reported among men (23.5%) and women (19.0%) in the Southern welfare states. In general, we found higher proportions of poor work-life balance among men than women across welfare states regimes. Regarding self-reported health, some gender differences were observed across welfare states. We observed the highest percentage of good self-reported health among men (84.8%) and women (87.2%) in the Liberal welfare states as compared to the other welfare states. In contrast, both working men (23.8%) and women (27.0%) in the CEE welfare states reported the highest poor self-reported health. In general, women reported slightly higher levels of education than men across the welfare states regimes. Also, the frequency of engaging in shift work was higher among women than men, particularly in the CEE welfare states regime (men: 24.8% vs women: 30.0%). Men were more likely to have their working time determined by themselves as compared to women in all the welfare states regimes. On the other hand, women frequently had their working time arrangements set by their companies. The results further revealed that men had long working hours and higher occupational status than women across all welfare states regimes.

**Table 1:** Descriptive statistics of women and men in the study (in means and percentages)

	Nordic		Conservative		Liberal		Southern		CEE	
Variables	Women (*n* = 1770)	Men (*n* = 1723)	Women (*n* = 4535)	Men (*n* = 4443)	Women (*n* = 1113)	Men (*n* = 1252)	Women (*n* = 3741)	Men (*n* = 4030)	Women (*n* = 5322)	Men (*n* = 4346)
	% or Mean	% or Mean	% or Mean	% or Mean	% or Mean	% or Mean	% or Mean	% or Mean	% or Mean	% or Mean
**Work-Life Balance**										
Good	86.89	85.61	85.05	81.99	84.64	80.99	80.99	76.48	84.74	80.9
Poor	13.11	14.39	14.95	18.01	15.36	19.01	19.01	23.52	15.26	19.1
**Self-Reported Health**										
Good	80.85	80.79	79.79	80.1	87.15	84.82	79.12	81.22	73.02	76.25
Poor	19.15	19.21	20.22	19.9	12.85	15.18	20.88	18.78	26.98	23.75
**Age**	44.59	44.49	42.73	42.82	42.28	43.12	41.59	42.26	43.44	42.42
**Household Size**	2.76	2.79	2.67	2.65	2.81	2.87	2.93	2.9	2.87	2.89
**Marital Status**										
Single or Widowed	31.58	28.09	38.17	33.2	39.8	30.75	38.23	35.93	31.75	30.67
Married or Cohabiting	68.42	71.91	61.83	66.8	60.2	69.25	61.77	64.07	68.25	69.33
**Living with a child**										
No	49.49	51.31	47.78	55.84	49.78	57.59	47.79	55.31	47.43	55.87
Yes	50.51	48.69	52.22	44.16	50.22	42.41	52.21	44.69	52.57	44.13
**Education**										
Early Childhood	0.28	–	0.24	0.32	0.72	0.24	0.94	1.36	–	–
Primary	0.56	1.22	2.4	2.32	0.81	2.32	7.11	7.64	0.23	0.46
Lower Secondary	4.63	8.47	10.39	11.01	20.84	27	17.94	21.34	7.22	8.86
Upper Secondary	27.85	39.12	46.46	50.51	20.4	20.77	27.69	30.65	48.37	58.19
Post Secondary Non-Tertiary	8.7	9.05	5.49	4.52	8.81	6.79	10.85	10.5	9.45	8.31
Short Cycle Tertiary	22.82	14.57	12.04	8.24	12.94	11.98	12.67	10.05	7.2	4.37
Bachelor	14.97	9.87	9.61	8.6	20.04	19.81	17.05	12.9	14.66	9.96
Master	18.7	16.42	12.13	13.19	14.38	9.98	4.7	4.27	12.34	9.18
Doctorate	1.47	1.28	1.23	1.31	1.08	1.12	1.04	1.29	0.54	0.67
**ISCO**										
Armed Forces Occupation	–	0.75	0.07	0.56	–	0.16	0.03	1.04	0.15	0.81
Managers	5.2	9	4.63	8.35	11.86	15.97	3.42	5.46	5.07	7.89
Professionals	36.05	25.48	21.35	17.17	25.07	18.37	20.85	14.07	22.66	10.26
Technicians and Associate	19.15	14.74	15.41	15.55	11.86	10.86	7.51	10.05	11.78	10.38
Clerical Support Workers	6.84	4.24	15.21	7.22	12.85	4.31	15.24	8.09	10.28	4.16
Service and Sales	22.49	11.14	27.32	12.6	28.84	13.26	29.72	18.06	28.18	13.09
Skilled Agricultural, Forestry and Fish	1.36	3.19	0.57	1.8	0.9	5.19	1.76	4.09	1.94	3.06
Craft and Related Trades	1.81	15.9	1.52	19.42	0.72	13.02	3.56	19.73	5.79	27.47
Plant and Machine Operators and Assembly	1.75	11.43	1.32	10.42	1.44	11.1	2.67	9.06	3.96	15.42
Elementary Occupation	5.37	4.12	12.59	6.69	6.47	7.75	15.24	10.35	10.18	7.46
**NACE**										
Agric	1.81	4.82	0.97	2.86	1.35	6.63	2.51	5.36	3.25	5.84
Industry	9.72	30.82	9.72	31.56	7.37	27.56	10.48	27.82	17.16	39.76
Service and Sales	81.19	59.61	79.96	61.33	84.64	61.74	73.96	61.74	72.57	50.21
Other	7.29	4.76	9.35	4.25	6.65	4.07	13.04	5.09	7.03	4.19
**Sector**										
Private	44.86	70.75	63.97	73.42	55.35	76.84	73.22	77.1	61.95	76.21
Public	50.96	23.51	25.12	18.79	37.47	19.49	20.69	16.9	33.62	19.1
Joint Private-Public	2.88	4.53	6.26	5.65	2.79	2.32	2.65	2.48	2.33	2.92
NGO	0.56	0.7	3.09	1.19	3.41	1.12	0.37	0.4	0.9	0.37
Other	0.73	0.5	1.57	0.95	0.99	0.24	3.07	3.13	1.2	1.4
**Shift Work**										
Yes	19.77	14.68	16.82	18.16	21.65	21.57	20.26	20.87	30.01	24.76
No	80.23	85.32	83.18	81.84	78.35	78.43	79.74	79.13	69.99	75.24
**Weekly Hours**										
30 h and Below	27.12	15.96	44.98	16.36	48.7	22.52	34.43	20.42	19.6	15.19
31–40 h	63.11	62.04	44.21	59.87	39.44	46.01	50.63	51.22	63.28	54.58
41–50 h	7.63	14.68	7.96	15.71	7.64	18.21	10.24	17.2	12.98	19.44
51h hrs	2.15	7.31	2.84	8.06	4.22	13.26	4.7	11.17	4.13	10.79
**Working Arrangement**										
Set by Company	36.16	32.97	51.03	52.31	62.08	51.84	69.07	64.27	72.27	68.43
Can Choose between Fixed Schedule	9.1	6.5	12.22	7.79	7.91	8.71	6.42	5.04	6.95	5.57
Flexible Time	42.88	42.77	24.17	24.02	20.58	22.28	11.31	9.9	11.01	11.9
Working Time is Determined by Self	11.86	17.76	12.59	15.89	9.43	17.17	9.77	20.79	9.77	14.1
**Type of Employment**										
Employee	93.95	87.35	90.76	87.19	90.66	77.4	85	76	90.83	85.07
Self-Employed	6.05	12.65	9.24	12.81	9.34	22.6	15	24	9.17	14.93

### Bivariate analysis

The results of the bivariate analysis between self-reported health and the measured variables are shown in Table 2. The bivariate analysis showed a significant association between work-life balance and self-reported health for both working men and women. Age was positively associated with self-reported health (men (*r* = 0.213) vs. women (*r* = 0.207)). We, however, found a negative but low correlation between household size and self-reported health for both men (*r* =  − 0.056 ) and women (*r* =  − 0.057). Marital status was found to be significantly associated with self-reported health for women, but not men. Meanwhile, a significant association was found between type of employment and self-reported health among men but not women. Overall, there were similar patterns of associations between measured variables and self-reported health among men and women.

**Table 2:** Relationship between self-reported health and other measured variables by gender

Variable	Self-Reported Health		Self-Reported health	
	Men		Women	
Education	(0.000)	***	(0.000)	***
Marital status	(0.123)	NS	(0.034)	**
Living with a child	(0.009)	***	(0.000)	***
Occupation	(0.000)	***	(0.000)	***
Type of Industry	(0.000)	***	(0.000)	***
Sector	(0.024)	**	(0.007)	***
Working arrangement	(0.013)	**	(0.020)	**
Weekly hours	(0.000)	***	(0.000)	***
Type of employment	(0.003)	***	(0.126)	NS
Shift work	(0.083)	*	(0.000)	***
Work-life balance	(0.000)	***	(0.000)	***
Age	0.213	***	0.207	***
Household size	−0.056	***	−0.0571	***

Significance level: ∗ ∗  ∗ *p* < 0.001, ∗ ∗  < 0.05, ∗ < 0.10

(): are the Pearson Chi-Square

Non bracket values are the Point Biserial correlation coefficients

*NS* not significant

The results for the VIF’s are shown in an additional file supplied in the table: S1 and S2. We compared the VIF’s of all the measured variables by gender. The maximum VIF for working men was 2.16, and the mean VIF was 1.45. For working women, the maximum VIF was 2.01, and the mean VIF was 1.33. The VIF estimated for men and women were quite similar. In fact, the estimated VIF’s were less than 2.5, which meets the threshold [78], for non-existence of multicollinearity.

### Multivariate analysis

Table 3 provides information on the multivariate logistic regression. After adjusting for socio-economic factors, working characteristics, and demographic characteristics, the results showed a significant association between poor work-life balance and poor self-reported health among working adults (aOR = 2.07; 95% CI: (1.93–2.23)). We also found a significant association between poor work-life balance and self-reported health for both men (aOR = 1.97; 95% CI: (1.78–2.18) and women (aOR = 2.21; 95% CI: (1.99–2.45)). However, the magnitude of the association differs slightly among men and women.

**Table 3:** Association between poor work-life balance and poor self-reported health among working men and women in Europe

	Men	Women	Total
Variable	aOR(95%CI)	aOR(95%CI)	aOR(95%CI)
Good Work-Life Balance (ref)			
Work-Life Conflict	1.97 (1.78–2.18)***	2.21 (1.99–2.45)***	2.07 (1.93–2.23)***

Significance level:***p < 0.001

CI: 95% confidence interval

aOR- Adjusted odds ratio

Odds ratios are adjusted for socio-economic positions, working characteristics, and demographic characteristics

### Multilevel logistic regression

Table 4 shows the country variation that exists in the relationship between poor self-reported health and work-life conflict. We used a two-stage multilevel logistic regression to assess variations that exist between welfare states regimes. After controlling for socio-economic positions, working characteristics, and demographic characteristics, the multilevel models showed a significant relationship between work-life conflict and poor self-reported. However, the magnitude of the associations differs slightly across welfare states regimes. For instance, we found the association to be slightly higher for women than men in the Nordic (men: aOR = 1.77; 95% CI: (1.26–2.47) vs women: aOR = 1.92; 95% CI: (1.37–2.69)), Liberal (men: aOR = 2.23; 95% CI: (1.55–3.21) vs women: aOR = 2.39; 95% CI: (1.51–3.78)), Southern (men: aOR = 1.65; 95% CI: (1.36–2.00) vs women: aOR = 2.02; 95% CI: (1.64–2.48)), and CEE welfare states (men: aOR = 1.91; 95% CI: (1.59–2.30) vs women: aOR = 2.29; 95% CI: (1.92–2.73)), but slightly higher for men than women in the Conservative welfare states (men: aOR = 2.62; 95% CI: (2.17–3.17) vs women: aOR = 2.42; 95% CI: (2.00–2.94)). While the largest odds between work-life conflict and poor self-reported health among men were found in the Conservative welfare states, the Liberal and the CEE welfare states, the smallest association was observed in the Southern European welfare states followed by the Nordic welfare states. Among women, the highest association was found in the Liberal and Conservative welfare states, while, the lowest association was observed in the Nordic welfare states regime.

**Table 4:** Between country variation of the associations between work-life conflict and self-reported health: Multi-level logistic regression of men and women in European welfare states

Variable	Nordic	Conservative	Liberal	Southern	CEE	Random Effects
	aOR(95%CI)	aOR(95%CI)	aOR(95%CI)	aOR(95%CI)	aOR(95%CI)	aOR(95%CI)
Men	1.77 (1.26–2.47)***	2.62 (2.17–3.17)***	2.23 (1.55–3.21)***	1.65 (1.362.00)***	1.91 (1.59–2.30)***	1.19 (0.96%)
Women	1.92 (1.37–2.69)***	2.42 (2.00–2.94)***	2.39 (1.51–3.78)***	2.02 (1.64–2.48)***	2.29 (1.92–2.73)***	1.29 (2.14%)

Significance level:***p < 0.001

Odds ratios are adjusted for socio-economic positions, working characteristics, and demographic characteristics

CI: 95% confidence interval

MOR: Median odds ratio

(VPC): Variance partition coefficient

Overall, we found small variation in the association between poor work-life balance and poor self-reported health between countries in Europe (men: MOR = 1.18 vs women: MOR = 1.29), and the percentage of variations were slightly higher for women (VPC = 2.07%) than men (VPC = 0.9%).

## Discussion

To the best of our knowledge, this is the first study to examine gender and welfare state regime differences in the relationship between work-life conflict and self-reported health among working adults with a comprehensive cross-country sample in Europe. The descriptive results revealed that working men in Europe had poor work-life balance than women in the Nordic, Conservative, Liberal, Southern, and CEE welfare states. We found the highest proportion of good work-life balance in the Nordic welfare states regime, while the highest proportion of poor work-life balance was found in the Southern European welfare states regime. More importantly, the result showed that poor work-life balance, as measured in the EWCS 2015 was strongly associated with poor self-reported health among working adults in Europe. However, the magnitude of the association was slightly higher for working women than men. Furthermore, we observed slight variations in the association between poor work-life balance and poor self-reported health across welfare states regimes in Europe. While the largest association between poor work-life balance and poor self-reported health for both men and women were observed in the Liberal welfare states and the Conservative welfare states, the smallest association was found in the Nordic and Southern welfare states.

### Work-life balance and health

Prior evidence that examined work-life conflict among men and women showed inconsistent findings [5, 33, 43–45]. Overall, our study found a higher frequency of poor work-life balance among men than women across welfare states regimes in Europe. This finding is consistent with a study conducted by Jansen et al. [82], who found evidence that men are most affected by work-life conflict as compared to women. While working men and women in the Nordic (men = 85.6% vs. women = 86.9%) and the conservative (men = 82.0% vs. women = 85.1%) welfare states reported the highest proportions of good work-life balance, the highest proportion of poor work-life balance among men and women were found in the Southern (men = 23.5% vs women = 19.0%), CEE (men = 19.1% vs women = 15.3%), and Liberal (men = 19.0% vs women = 15.4%) welfare states. These findings were partly in agreement with the findings of the 2010 European Working Conditions Survey by Lunau et al. [5]. They found a higher prevalence of poor work-life balance among working men and women in the Southern, CEE, and Former Soviet Union welfare states. Juxtaposing our results to the findings by Luanu et al. [5] revealed that poor work-life balance among employees in Europe appears to have reduced over time, perhaps, due to improvement in working conditions for employees [83].

Our results further revealed a strong association between poor work-life balance and poor self-reported health among working adults in Europe. This finding is in congruence with other studies that found a negative association between poor work-life balance and self-reported health [5, 41, 66, 84]. For instance, a systematic review by Allen, Herst, et al. [66] suggest that poor work-life balance was associated with poor health outcomes including psychological strain, depression, burnout, stress, and substance abuse. Likewise, research conducted among workers in Korea indicated that poor work-life balance was positively associated with poor health outcomes such as fatigue, general health, mental health, sickness absenteeism, musculoskeletal diseases, and work-related risks to health and safety [41]. This adverse relationship can partly be explained by the multiple role engagement and overload of demands and responsibilities among working adults [43].

Regarding gender, while some studies suggest that there is no difference in the relationship between work-life conflict and poor self-reported health among men and women [5, 28], few studies noted higher adverse health outcomes among women than men [25–27]. Our findings from the multivariate analysis indicated that there is a negative relationship between work-life conflict and self-reported health among men and women in contemporary welfare states in Europe, consistent with prior studies [5, 25–27]. However, there is a slight difference in the strength and magnitude of the association, where the association is slightly higher among women than men. This outcome has been attributed to behavioural norms and societal expectations for men and women [33], and differential exposure to multiple role engagement and overloads, pressures of family, work demands, and social commitment [43]. While women are expected to devote more time to family roles such as housekeeping, elderly care, and child care, men are expected to engage more in paid work activities [10, 34–36, 38, 85]. This finding was evident in our study as the proportion of weekly working hours was higher among men than women, even though women reported higher levels of education than men in Europe. This unequal distribution of work-related activities (i.e. paid work and housework) may partly explain the gender work-life “imbalance” [25, 26] and adverse health outcomes, especially among women [27].

In terms of the variations between welfare states, the two-stage multilevel logistic regression showed a higher magnitude in the association between poor work-life balance and poor self-reported health for both men and women in the Liberal welfare states regime, where there is a strong male breadwinner tradition, minimal social policies, and poorly regulated labour market [58, 59]. Meanwhile, the magnitude of the association for both genders in the Conservative welfare states regime was slightly higher than the Nordic, Southern, and the CEE welfare states. Women had higher associations between poor work-life balance and poor self-reported health in this welfare states than men. We speculate that the presence of weak employment regulation and weak unions may allow for strict managerial control, which may increase job pressure and job insecurity [86]. In addition, the weak provision of social benefits for ‘child and elderly care’ [58] may exert pressure on women than men when combining care, household activities, and work demands [64].

Similar to other studies [13, 64, 87], we found a negative relationship between poor work-life balance and self-reported health for both men and women in the Conservative welfare states regime which is characterised by traditional breadwinner model and strong labour laws which regulate the labour market [53, 74]. Surprisingly, working men and women in the Conservative welfare states regime had the highest magnitude regarding the association between poor work-life balance and poor self-reported health. There is some evidence that women who live in Conservative welfare states report poorer health status than men due to poor work-life balance [87]. In contrast, our findings suggest a slightly higher magnitude in the association for men than women. These gender differences may probably be due to temporary contracts [88], as well as part-time employment for women as compared to men [60], which may provide women with more time resources to deal with competing demands than men [12, 13]. Further, we found a lower association between poor work-life balance and poor self-reported health for working adults in the Nordic welfare states regime than the Conservative, Liberal, and CEE welfare states. This may partly be attributed to the generous and encompassing social policies including child care for pre-school, universal health care, elderly care, and long parental leave days that exist in the Nordic welfare states [53, 55–57], which may contribute to lower levels of work-life conflict and consequently better self-reported health [5, 12, 13]. However, poor work- life balance was slightly associated with poor self-reported health among women than men in the Nordic welfare states. The slight gender difference in the association may be related to the so-called “parallel ideals” that exist in the Nordic countries where equality is important; yet societal and cultural expectation of women as caregivers still exist [12, 64, 89]. Surprisingly, we found weaker associations between poor work-life balance and poor self-reported health among working adults in the Southern European welfare states as compared with Conservative and Liberal welfare states, especially among men. This is very striking considering the fact that Southern welfare states are characterized by minimal social welfare benefits than the conservative welfare state [64]. In view of the above discussion, and based on the median odds ratios (MOR) and the variance partition coefficients (VPC) that were estimated in the two-stage multi-level logistic regression, our findings suggested that the effect size of work-life balance on health status may vary between welfare states in Europe, particularly, among women than men. Nonetheless, the magnitude of the variation between welfare state regimes that was identified in our study were quite marginal.

In order to address the issue of work-life conflict among working adults in the welfare states in Europe, countries must design, and implement effective industrial relation laws, legislation, and policies that can effectively protect the health and safety of working adults [90, 91]. The existing laws and regulation can also be effectively enforced through agencies, where labour inspectorate can oversee the enforcement of existing labour protection laws and work-life policies such as work time arrangement, paid parental leave days, and child and elderly care. Furthermore, welfare states should establish strong state institutions and judicial systems to serve as mediators that can assist workers and employers in the resolution of disputes [90]. For example, there must be well-functioning labour court, special tribunal and arbitration system which is easily accessible for workers and employers to address disputes pertaining to work and family life [90]. Finally, governments must encourage and strengthen frequent tripartite negotiation between welfare states and its representatives, employers, trade unions, and other stakeholders to dialogue on the implementation and sustainability of family-friendly policies [90, 91].

### Limitation and strength

Although the findings of this research are in line with previous research and empirical reviews, there are some conceptual limitations that need to be addressed. Firstly, the measure of work- life balance by the EWCS 2015 was assessed by using only one question on “whether working hours fit in with family or social commitments”. Although work-family fit serves as an important proxy in dealing with issues of work-life balance, it lacks the theoretical basis in describing how the dimensions of work-life conflict and facilitation operate together in shaping the individual and organization [92]. Greenhaus and Beutell [3] noted that work-life conflict can be measured through different dimensions such as time, strain, and behaviour based conflict. However, the EWCS 2015 only captured the time conflict dimension and not strain and behaviour-based conflict. As suggested by Choi and Kim [41], future studies should combine both the work-life balance measurement in the EWCS to the OECD measurement of work-life balance to form one comprehensive question that includes all the dimensions. Secondly, we used self–reported health as the outcome variable. This subjective measure has been linked with heterogeneity problems [93, 94], where people living in different locations, and with different socio-economic status, family demographic status, and gender may adopt different thresholds in assessing their health [94]. Nevertheless, self-reported health has been shown to be an accurate measure and a strong predictor for mortality [95]. Thirdly, our findings were based on cross-sectional data, which makes it difficult to make definitive conclusions on the direction of the relationship between work-life balance and health status among employees [96]. Notwithstanding these limitations, this study is the first to provide a comprehensive overview of the relationship between work-life conflict and self-reported health according to welfare state regime typology.

## Conclusion

We conclude that poor work-life balance is associated with poor self-reported health among working adults, particularly among working women than men in Europe. However, the magnitude and strength of these associations slightly differ across countries in different welfare states regimes. This study thus serves as the baseline for policymakers and stakeholders to fully understand the need to help reduce pressing demands from life domains. Organizations must also create good working atmosphere and flexible working time to deal with issues of jobs strain in order to reduce health problems.

## Supplementary information

**Additional file 1: Table S1** Variance Inflation Factor (VIF) of measured variables by men. **Table S2** Variance Inflation Factor (VIF) of measured variables by women.

## Data Availability

The data used for this study comes from European Foundation for the Improvement of Living and Working Conditions. Detailed information on the survey design and characteristics are provided on the https://www.eurofound.europa.eu/surveys/european-working-conditions-surveys/sixth-european-working-conditions-survey-2015 homepage.
