# Supplementary material for: Work-life balance and self-reported health among working adults in Europe: a gender and welfare state regime comparative analysis
Source: BMC Public Health. 2020 Jul 16;20:1052. doi: 10.1186/s12889-020-09139-w (PMC7364652; doi:10.1186/s12889-020-09139-w)
Supplement: Supplementary file 1 — Additional file 1: Table S1 Variance Inflation Factor (VIF) of measured variables by men. Table S2 Variance Inflation Factor (VIF) of measured variables by women. [file 12889_2020_9139_MOESM1_ESM.docx]

**Appendix**

Table S1 Variance Inflation Factor (VIF) of measured variables by men

| Variable | VIF |
| --- | --- |
| Child | 2.16 |
| Household size | 2.03 |
| Working arrangement | 1.76 |
| Employment Type | 1.65 |
| Occupation | 1.56 |
| Partner | 1.54 |
| Education | 1.46 |
| Age | 1.23 |
| Type of industry | 1.13 |
| Weekly hour | 1.12 |
| Shift work | 1.10 |
| Work-life balance | 1.07 |
| sector | 1.05 |
| Mean VIF | 1.45 |

Table S2 Variance Inflation Factor (VIF) of measured variables by women

| Variable | VIF |
| --- | --- |
| Household size | 2.01 |
| Child | 1.72 |
| Education | 1.50 |
| Occupation | 1.50 |
| Working arrangement | 1.43 |
| Employment type | 1.41 |
| Partner | 1.23 |
| Age | 1.14 |
| Weekly hour | 1.10 |
| Shift work | 1.08 |
| Sector | 1.07 |
| Work-life balance | 1.06 |
| Type of industry | 1.04 |
| Mean VIF | 1.33 |
